# Multi-cancer computational analysis reveals invasion-associated variant of desmoplastic reaction involving INHBA, THBS2 and COL11A1

**DOI:** 10.1186/1755-8794-3-51

**Published:** 2010-11-03

**Authors:** Hoon Kim, John Watkinson, Vinay Varadan, Dimitris Anastassiou

**Affiliations:** 1 Center for Computational Biology and Bioinformatics and Department of Electrical Engineering, Columbia University, New York, NY, USA; 2 Philips Research North America, Briarcliff Manor, NY, USA

## Abstract

**Background:**

Despite extensive research, the details of the biological mechanisms by which cancer cells acquire motility and invasiveness are largely unknown. This study identifies an invasion associated gene signature shedding light on these mechanisms.

**Methods:**

We analyze data from multiple cancers using a novel computational method identifying sets of genes whose coordinated overexpression indicates the presence of a particular phenotype, in this case high-stage cancer.

**Results:**

We conclude that there is one shared "core" metastasis-associated gene expression signature corresponding to a specific variant of stromal desmoplastic reaction, present in a large subset of samples that have exceeded a threshold of invasive transition specific to each cancer, indicating that the corresponding biological mechanism is triggered at that point. For example this threshold is reached at stage IIIc in ovarian cancer and at stage II in colorectal cancer. Therefore, its presence indicates that the corresponding stage has been reached. It has several features, such as coordinated overexpression of particular collagens, mainly *COL11A1 *and other genes, mainly *THBS2 *and *INHBA*. The composition of the overexpressed genes indicates invasion-facilitating altered proteolysis in the extracellular matrix. The prominent presence in the signature of INHBA in all cancers strongly suggests a biological mechanism centered on activin A induced TGF-β signaling, because activin A is a member of the TGF-β superfamily consisting of an INHBA homodimer. Furthermore, we establish that the signature is predictive of neoadjuvant therapy response in at least one breast cancer data set.

**Conclusions:**

Therefore, these results can be used for developing high specificity biomarkers sensing cancer invasion and predicting response to neoadjuvant therapy, as well as potential multi-cancer metastasis inhibiting therapeutics targeting the corresponding biological mechanism.

## Background

There is currently great interest in identifying the biological mechanisms for the acquisition of motility and invasiveness in cancer. It has been hypothesized [1,2] that they often involve some form of epithelial-mesenchymal transition (EMT), significant involvement of the tumor microenvironment [3,4] and the presence of activated fibroblasts in the "reactive" desmoplastic stroma of tumors, referred to as "cancer associated fibroblasts" (CAFs) [5,6].

A study [7] of serous papillary ovarian carcinomas, comparing the gene expression profiles of primary vs. omental metastatic tumors, identified 156 differentially expressed genes. To investigate the significance of these genes in an independent rich data set we performed hierarchical clustering, using only these genes, on The Cancer Genome Atlas (TCGA) gene expression data set consisting of 377 ovarian cancer samples containing staging information. The resulting heat map revealed a prominent block of about 100 highly overexpressed genes in 94 samples (Additional file 1). Remarkably, we found that none of the 41 samples from tumors of stages IIIb and below were among the 94 samples. This cannot be due to chance (p = 4 × 10^-6^), leading to the hypothesis that coordinated overexpression of these genes implies that the tumor has progressed to at least stage IIIc.

To further validate this hypothesis and test if similar versions apply to other cancers, we developed a computational technique identifying in an unbiased manner coordinatedly overexpressed genes associated with a phenotype (such as transition to a particular stage). Our results consistently rediscover the same "core" signature of overexpressed genes, confirming the hypothesis. We found that the signature is present in multiple cancers, each of which has its own features involving additional genes, but the core signature is common.

As evidenced by the presence of *FAP *(fibroblast activation protein) and *ACTA2 *(actin, alpha 2, also known as *α-SMA*, alpha-smooth muscle actin) in the set of overexpressed genes, the signature suggests a variant of stromal desmoplastic reaction. As further evidence, it is known ([6], p. 546) that activated fibroblasts (myofibroblasts) construct the desmoplastic stroma through the secretion of large amounts of collagen, fibronectin (FN1) and proteoglycans, and they secrete various proteases such as urokinase plasminogen activator (PLAU) and matrix metalloproteinases (MMPs). This list precisely describes most of the genes appearing in the signature, and its precise composition (e.g., having COL11A1 as the prominent collagen, MMP11 as the prominent metalloproteinase, and CDH11 as the prominent cadherin) points to a particular variant of CAFs, to which we refer as "metastasis associated fibroblasts" (MAFs). Indeed, the resulting proteolytic remodeling at the invasive edge of the tumor is thought to facilitate the initial invasive stage of the metastatic process by "excavating passageways" ([6], p. 621) through the extracellular matrix for the cancer cells to go through. Accordingly, in the following we refer to the corresponding gene expression signature and biological mechanism as "the MAF signature" and "the MAF mechanism," respectively.

## Methods

### List of data sets

The list of data sets in the paper is given in Table 1. They were identified by searching for rich data sets focused on a specific cancer in two public databases, The Cancer Genome Atlas and the Gene Expression Omnibus data depository. Furthermore, for the data sets initially used to infer the gene signature we required that they have well annotated staging information associated with the samples and that they contain a significant number of samples in both lower and higher stages so that we could compare the expression profiles across stages.

**Table 1:** Lists of Data sets in the paper

Data set name	Source Site	GEO Accession	Affymetrix platform	Sample size
TCGA Ovarian Cancer	The Cancer Genome Atlas		HT_HG-U133A^**a**^	377
CCR Ovarian Cancer	Gene Expression Omnibus	GSE9891	HG-U133_Plus_2^**b**^	285
CCR Colon Cancer	Gene Expression Omnibus	GSE14333	HG-U133_Plus_2	290
Moffitt Colon Cancer	Gene Expression Omnibus	GSE17536	HG-U133_Plus_2	177
Singapore Gastric Cancer	Gene Expression Omnibus	GSE15459	HG-U133_Plus_2	200
CCR Breast Cancer	Gene Expression Omnibus	GSE7390	HG-U133A^**c**^	198
Wang Breast Cancer	Gene Expression Omnibus	GSE2034	HG-U133A	286
Samsung Lung Cancer	Gene Expression Omnibus	GSE8894	HG-U133_Plus_2	138
Bild Lung Cancer	Gene Expression Omnibus	GSE3141	HG-U133_Plus_2	111
Neuroblastoma tumor	Gene Expression Omnibus	GSE3960	HG_U95Av2	102
Neoadjuvant Breast Cancer	Gene Expression Omnibus	GSE4779	U133_X3P	[65] 102^e^
Postmortem - normal	Gene Expression Omnibus	GSE3526	HG-U133_Plus_2	353
Human body index - normal	Gene Expression Omnibus	GSE7307	HG-U133_Plus_2	504 [677]^f^

^**a**^Affymetrix HT Human Genome U133A Array

^**b**^Affymetrix Human Genome U133 Plus 2.0

^**c**^Affymetrix Human Genome U133A

^d^270 out of 341 samples are tumor samples.

^e^65 out of 102 are ER-negative samples.

^f^504 out of 677 samples are normal samples.

### Extreme Value Association (EVA)

Since we aim to discover a set of genes that are coordinately overexpressed only in a subset of the "high stage" samples, we developed a special measure of association between the gene and the binary ("high stage" vs. "low stage") phenotype that naturally fits this description, ignoring the expression levels of the genes outside the region of overexpression, which we call "extreme value association" (EVA). The same measure can identify coordinately silenced genes, but we did not find any such genes across various cancer types.

**Figure 1:**
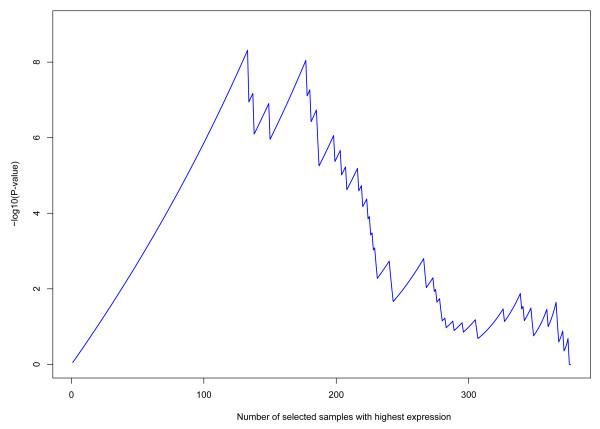
**Evaluation of the EVA metric for gene *COL11A1 *in the TCGA ovarian cancer data set**.

The EVA metric is the minimum p-value of biased partitions over all subsets of samples with highest expression values of the gene. In other words, suppose that there are totally *M *samples, out of which *N *are "low stage" and *M *- *N *are "high stage," and we select the *m *samples with the highest gene expression values, out of which *n *are "low stage" and *m *- *n *are "high stage." Under the assumption that gene expression values are uncorrelated with the phenotype, the probability that there will be at most *n *"low stage" samples among the selected *m *samples is given by the cumulative hypergeometric probability *h*(*x *≤ *n*; *M*, *N*, *m*). The EVA metric is then defined as equal to -log_10 _of the minimum of these probabilities over all possible choices of *m*, in which *m *ranges from 1 to *M *(note that *n *depends on *m*). For example, assume that there are 250 high-stage samples and 50 low-stage samples for a total of 300 samples. Furthermore, assume that the 100 samples with the highest values of a particular gene contain 99 high-stage samples and one low stage sample. In that case, *h*(*x *≤ 1;300,50,100) can be evaluated using the MATLAB function hypercdf (1,300,50,100) = 5 × 10^-9^, resulting in the EVA metric for that gene of at least -log_10_(5 × 10^-9^) = 8.3. If the 101^th ^sample is also high-stage, then the EVA metric of the gene will be even higher. Note that, once the highest value is reached, the sorting arrangement of the remaining samples is irrelevant, reflecting the hypothesis that only the extreme values are associated with the phenotype. Figure 1 shows the values of the cumulative hypergeometric probability for the *COL11A1 *gene using the TCGA ovarian cancer data set and the staging threshold for the definition of the binary phenotype set between IIIb and IIIc. The maximum (8.31) occurs when *m *= 133.

We then developed a mechanistic and unbiased (only dependent on the phenotype) algorithm, which, when given a gene expression data set for a number of samples labeled "high stage" or "low stage," leads to a selection of genes that are coordinatedly overexpressed only in high-stage samples, ignoring the effect of the rest, thus precisely reflecting the observed phenomenon. We first select the top 100 genes that rank highest according to the EVA metric criterion using a mixture analysis (selecting the minimum p-value) of both overexpressed and silenced genes. Using this set of genes only, we perform k-means clustering with gap statistic [8]. At that step, if indeed the genes are coordinately overexpressed, they will align well in the heat map. This leads to the selection of the samples belonging to the cluster most associated with the high/low stage phenotype - call this the set of "EVA-based samples." Next, we define a "clean" MAF phenotype, contrasting the samples that are: (a) both "EVA based" and "high-stage" against (b) the samples that are both "non EVA-based" and "low stage." If the number of samples is sufficiently large, this "clean" phenotype provides the sharpest way by which we can identify the genes that are most associated with the observed phenomenon of metastasis-associated coordinated overexpression. We then rank the genes and compute their multiple-test-corrected p-values using a Wilcoxon rank-sum test using the "clean" phenotype and select the genes for which p < 10^-3 ^after Bonferroni correction. Finally, we find the intersection of these selected gene sets over all cancer expression data sets and rank them in terms of fold change.

For a data set with *n *samples and *m *genes, the EVA algorithm computes cumulative hypergeometric distribution probabilities *nm *times. This can be computationally intensive, so we devised a low-complexity implementation algorithm described in Additional file 2.

### Mutual Information and Synergy

Assuming that two variables, such as the expression levels of two genes *G*_1 _and *G*_2_, are governed by a joint probability density *p*_12 _with corresponding marginals *p*_1 _and *p*_2 _and using simplified notation, the mutual information *I*(*G*_1 _; *G*_2_) is a general measure of correlation and is defined as the expected value $E\left\{\log\frac{p_{12}}{p_{1}p_{2}}\right\}$. The synergy of two variables *G*_1_, *G*_2_, with respect to a third variable *G*_3 _is [9] equal to *I*(*G*_1_, *G*_2_; *G*_3_) - [*I*(*G*_1_; *G*_3_) + *I*(*G*_2_; *G*_3_)], i.e., the part of the association of the pair *G*_1_, *G*_2 _with *G*_3 _that is purely due to a synergistic cooperation between *G*_1 _and *G*_2 _(the "whole" minus the sum of the "parts"). We used a 3^rd ^order B-spline-based mutual information estimator dividing the observation space into six equally spaced bins in each dimension.

### P-value estimation for mutual information and synergy

We applied a permutation-based approach accounting for multiple test correction: We did 100 permutation experiments of the class labels, saving the corresponding 100 highest values after doing exhaustive search in each permutation experiment. Using the set of these 100 highest value scores, we obtained the maximum likelihood estimates of the location parameter and the scale parameter of the Gumbel (type-I extreme value) distribution, resulting in a cumulative density function *F*. The p-value of an actual score *x*_0 _is then 1 - *F*(*x*_0_) under the null hypothesis of no association with phenotype. Similarly, for the synergistic pair, we found the top-scoring synergy in 100 data sets that were identical to the original except that the phenotype values were randomly permuted on each, and the top permuted synergy scores were modeled, as above, with the Gumbel distribution.

## Results

### Identification of MAF signature genes from staging information in four data sets

We performed the EVA algorithm on four rich gene expression data sets, two from ovarian cancer, the one from TCGA and another one [10], and two from colorectal cancer [11,12] accompanied by staging information. We performed the algorithm multiple times using the different possible cutoff thresholds defining the phenotype, finding that, in all cases, it is defined as exceeding stage IIIb in each of the ovarian data sets and stage I in each of the colorectal data sets. Interestingly, several among the "metastasis-associated genes" identified in [7] as present in omental metastasis of ovarian cancer were also identified in [10] as belonging to a subtype of ovarian cancer characterized by extensive desmoplasia, which contains the MAF signature.

Remarkably, we found that there were 137 genes (listed in Additional file 3), each of which had Bonferroni-corrected p < 10^-3 ^in *all four *data sets. Table 2 shows a list of these genes with average log fold change greater than 2. The top ranked gene was *COL11A1 *(probe 37892_at). Again, these genes were found purely as a result of their association with the staging phenotype in all four cancers. Gene Ontology enrichment testing of these genes identified cell adhesion, extracellular matrix and collagen fibril organization. It turns out that use of other standard correlation measures instead of the EVA measure in the same algorithms leads to the same results, because the overall correlation of these genes with the phenotype is strong merely as a result of the genes' overexpression in some high-stage samples alone. The EVA method has the additional advantage of providing an estimate of the size of that subset of high-stage samples.

**Table 2:** Top-ranked genes associated with carcinoma stage in four ovarian and colorectal cancers

**Probe Set**^**a**^	Gene	Log FC
37892_at	*COL11A1*	3.94
217428_s_at	*COL10A1*	3.55
204320_at	*COL11A1*	3.39
210809_s_at	*POSTN*	3.14
206439_at	*EPYC*	3.12
219087_at	*ASPN*	2.99
205941_s_at	*COL10A1*	2.88
203083_at	*THBS2*	2.81
209955_s_at	*FAP*	2.73
215446_s_at	*LOX*	2.63
204051_s_at	*SFRP4*	2.53
210511_s_at	*INHBA*	2.52
215646_s_at	*VCAN*	2.50
218469_at	*GREM1*	2.48
209758_s_at	*MFAP5*	2.42
218468_s_at	*GREM1*	2.35
212353_at	*SULF1*	2.34
221730_at	*COL5A2*	2.34
211571_s_at	*VCAN*	2.33
204619_s_at	*VCAN*	2.33
205713_s_at	*COMP*	2.31
221731_x_at	*VCAN*	2.27
204620_s_at	*VCAN*	2.26
201150_s_at	*TIMP3*	2.25
221729_at	*COL5A2*	2.24
212354_at	*SULF1*	2.23
212489_at	*COL5A1*	2.22
213790_at	*ADAM12*	2.21
212488_at	*COL5A1*	2.20
201147_s_at	*TIMP3*	2.19
204457_s_at	*GAS1*	2.17
206026_s_at	*TNFAIP6*	2.14
202952_s_at	*ADAM12*	2.12
202766_s_at	*FBN1*	2.08
212344_at	*SULF1*	2.07
202311_s_at	*COL1A1*	2.05
209335_at	*DCN*	2.01

^**a**^Affymetrix probe sets

All genes have Bonferroni corrected p < 10^-3 ^in each of the four data sets.

We then did an extensive literature search aimed at identifying other studies in which at least some of these genes were identified as differentially expressed in various stages of other cancers. We even scrutinized studies in which none of the genes were mentioned in the main text, by looking at their supplementary data and re-ranking particular columns of genes in terms of their fold changes, from genes containing numerous genes. Although most of our results were negative, we were able to produce cancer gene lists with striking similarity (Table 3) to our own list (Table 2) in three studies of breast [13], gastric [14] and pancreatic [15] cancer.

**Table 3:** Gene lists produced from information provided in the corresponding papers for other cancers

**Breast Cancer, Shuetz et al**^**a**^			**Gastric cancer, Vecchi et al**^**b**^			**Pancreatic cancer, Badea et al**^**c**^		
**Probe Set**^**d**^	**Gene Symbol**	**Log FC**	**Probe Set**^**d**^	**Gene Symbol**	**Log FC**	**Probe Set**^**d**^	**Gene Symbol**	**Log FC**

37892_at	*COL11A1*	6.50	37892_at	*COL11A1*	4.26	227140_at	*INHBA*	5.15
204320_at	*COL11A1*	4.08	217428_s_at	*COL10A1*	4.15	217428_s_at	*COL10A1*	5.00
217428_s_at	*COL10A1*	4.07	209955_s_at	*FAP*	3.40	1555778_a_at	*POSTN*	4.92
213764_s_at	*MFAP5*	3.73	235458_at	*HAVCR2*	3.30	212353_at	*SULF1*	4.63
213909_at	*LRRC15*	3.61	204320_at	*COL11A1*	3.28	226237_at	*COL8A1*	4.60
205941_s_at	*COL10A1*	3.52	205941_s_at	*COL10A1*	3.21	37892_at	*COL11A1*	4.40
210511_s_at	*INHBA*	3.44	204052_s_at	*SFRP4*	2.90	225681_at	*CTHRC1*	4.38
202766_s_at	*FBN1*	3.43	226930_at	*FNDC1*	2.85	202311_s_at	*COL1A1*	4.12
212353_at	*SULF1*	3.35	227140_at	*INHBA*	2.77	203083_at	*THBS2*	3.97
218468_s_at	*GREM1*	3.35	209875_s_at	*SPP1*	2.77	227566_at	*HNT*	3.90
215446_s_at	*LOX*	3.22	205422_s_at	*ITGBL1*	2.63	204619_s_at	*CSPG2*	3.87
221730_at	*COL5A2*	3.22	226311_at	*---*	2.63	229802_at	*WISP1*	3.80
218469_at	*GREM1*	3.20	222288_at	*---*	2.62	212464_s_at	*FN1*	3.69
212489_at	*COL5A1*	3.08	231993_at	*---*	2.50	205713_s_at	*COMP*	3.53
203083_at	*THBS2*	2.99	226237_at	*COL8A1*	2.48	221729_at	*COL5A2*	3.38
201505_at	*LAMB1*	2.97	223122_s_at	*SFRP2*	2.47	209955_s_at	*FAP*	3.37
209955_s_at	*FAP*	2.96	210511_s_at	*INHBA*	2.43	229218_at	*COL1A2*	3.16
209758_s_at	*MFAP5*	2.92	203819_s_at	*IMP-3*	2.39	209016_s_at	*KRT7*	3.13
202363_at	*SPOCK*	2.91	212464_s_at	*FN1*	2.36	210004_at	*OLR1*	3.03
213241_at	*NY-REN-58*	2.90	212353_at	*SULF1*	2.35	219773_at	*NOX4*	3.02
205479_s_at	*PLAU*	2.89	227995_at	*---*	2.34	218804_at	*TMEM16A*	2.90
206584_at	*LY96*	2.88	225681_at	*CTHRC1*	2.30	238617_at	*---*	2.87
204475_at	*MMP1*	2.83	204457_s_at	*GAS1*	2.27	224694_at	*ANTXR1*	2.82
202952_s_at	*ADAM12*	2.83	216442_x_at	*FN1*	2.25	228481_at	*COX7A1*	2.77
201792_at	*AEBP1*	2.81	223121_s_at	*SFRP2*	2.23	226311_at	*ADAMTS2*	2.76
204114_at	*NID2*	2.81	211719_x_at	*FN1*	2.23	201792_at	*AEBP1*	2.68
213790_at	*ADAM12*	2.80	204776_at	*THBS4*	2.18	203021_at	*SLPI*	2.65
209156_s_at	*COL6A2*	2.77	210495_x_at	*FN1*	2.15	227314_at	*ITGA2*	2.58
219179_at	*DACT1*	2.74	202800_at	*SLC1A3*	2.13	205499_at	*SRPX2*	2.44
212488_at	*COL5A1*	2.73	214927_at	*---*	2.11	226997_at	*---*	2.41
219087_at	*ASPN*	2.73	212354_at	*SULF1*	2.09	219179_at	*DACT1*	2.36
204619_s_at	*CSPG2*	2.70	238654_at	*LOC147645*	2.06	203570_at	*LOXL1*	2.30
204337_at	*RGS4*	2.69	213943_at	*TWIST1*	2.06	201850_at	*CAPG*	2.25
204620_s_at	*CSPG2*	2.69	236028_at	*IBSP*	2.05	222449_at	*TMEPAI*	2.19
212354_at	*SULF1*	2.68	228481_at	*POSTN*	2.00	227276_at	*PLXDC2*	2.16

^**a**^Breast cancer list indicates genes overexpressed in invasive ductal carcinoma vs. ductal carcinoma in situ.

^**b**^Gastric cancer list indicates genes overexpressed in early gastric cancer vs. advanced gastric cancer.

^**c**^Pancreatic cancer list indicates genes overexpressed in pancreatic ductal adenocarcinoma vs. normal pancreatic tissue.

^**d**^Affymetrix probe sets

Specifically, a breast cancer study [13] comparing ductal carcinomas in situ (DCIS) with invasive ductal carcinoma (IDC) had a list of genes upregulated in IDC that had similarities to those we had identified, and the top-ranked gene was again *COL11A1 *(probe 37892_at) with log fold change of 6.50, while the next highest (4.08) corresponded to another probe of *COL11A1*, followed by a probe of *COL10A1*. Second, a study [14] comparing early gastric cancer (EGC) with advanced gastric cancer (AGC) - roughly separating stages I and II - also identified a similar differentially expressed gene list of which again *COL11A1 *(probe 37892_at) was at the top (log fold change: 4.26) followed by *COL10A1 *and *FAP*. Third, a study of pancreatic ductal adenocarcinoma [15] identified a list of genes overexpressed in whole tumor tissue versus normal pancreatic tissue, in which *COL11A1 *(probe 37892_at) is again prominent and the top entry (log fold change 5.15) was *INHBA*, supportive of our hypothesis of activin A induced TGF-β signaling. The presence of the MAF signature in the latter study indicates that pancreatic cancer had already become invasive in most cases before the biopsy. The prominent desmoplastic reaction in pancreatic cancers (which contains the MAF signature) has recently been increasingly recognized as a "foe" [16] that could lead to new therapeutic strategies targeting stromal cells to inhibit cancer. Finally, we realized that *COL11A1 *has been identified as a potential metastasis-associated gene in other types of cancer as well, such as in lung [17], and oral cavity [18], suggesting that the MAF signature may be present in a subset of high stage samples of most if not all epithelial cancers.

### Using COL11A1 as proxy for the MAF signature in the absence of staging information

In those cases as well as in our own findings, there was prominent presence of *COL11A1 *(probe 37892_at). This remarkable consistent strong association of COL11A1 with the staging phenotype (specific to each cancer type) suggests that it could be used as a "proxy" of the MAF signature. This would allow us to improve on the gene list of Table 2 by making use of numerous publicly available gene expression data sets of cancers of many types, even without any staging information, as long as the MAF signature is present in a sizeable subset of them, aiming at finding the "intersection" of the associated factors in these sets, revealing the "core" of the MAF biological mechanism.

As a first step for this task, we identified the genes that are consistently highest associated with *COL11A1*. Additional file 4 shows a listing of genes in nine cancer data sets, while Table 4 shows an aggregate list of the top 100 genes ranked in terms of their association (mutual information) with *COL11A1*. The list is similar to the phenotype-based gene ranking (Table 2). In addition to a few collagens of type XI, X, V, and I, the top ranked genes are thrombospondin-2 (*THBS2*), inhibin beta A (*INHBA*), leucine rich repeat containing 15 (*LRRC15*), versican (*VCAN*), fibroblast activation protein (*FAP*), and matrix metallopeptidase 11 (*MMP11*) aka stromelysin 3. The presence of *FAP *indicates a general desmoplastic reaction and is not, by itself, sufficient for inferring the MAF signature.

**Table 4:** Aggregate list of top genes associated with *COL11A1*

**Probe Set**^**a**^	*Gene*	MI			
37892_at	*COL11A1*	0.727	218469_at	*GREM1*	0.231
204320_at	*COL11A1*	0.640	201261_x_at	*BGN*	0.228
203083_at	*THBS2*	0.418	213125_at	*OLFML2B*	0.228
217428_s_at	*COL10A1*	0.386	201744_s_at	*LUM*	0.228
205941_s_at	*COL10A1*	0.373	202998_s_at	*ENTPD4*	0.223
221729_at	*COL5A2*	0.370	201438_at	*COL6A3*	0.223
210511_s_at	*INHBA*	0.368	212344_at	*SULF1*	0.222
221730_at	*COL5A2*	0.367	209596_at	*MXRA5*	0.221
213909_at	*LRRC15*	0.342	213764_s_at	*MFAP5*	0.221
212488_at	*COL5A1*	0.337	204589_at	*NUAK1*	0.216
204619_s_at	*VCAN*	0.326	217762_s_at	*RAB31*	0.216
209955_s_at	*FAP*	0.323	213905_x_at	*BGN*	0.214
202311_s_at	*COL1A1*	0.322	201150_s_at	*TIMP3*	0.214
221731_x_at	*VCAN*	0.320	221541_at	*CRISPLD2*	0.214
203878_s_at	*MMP11*	0.319	217763_s_at	*RAB31*	0.212
212489_at	*COL5A1*	0.318	217430_x_at	*COL1A1*	0.212
210809_s_at	*POSTN*	0.315	205422_s_at	*ITGBL1*	0.210
202310_s_at	*COL1A1*	0.314	201147_s_at	*TIMP3*	0.209
204620_s_at	*VCAN*	0.312	218468_s_at	*GREM1*	0.209
202404_s_at	*COL1A2*	0.304	217764_s_at	*RAB31*	0.208
202952_s_at	*ADAM12*	0.300	213765_at	*MFAP5*	0.206
213790_at	*ADAM12*	0.297	211668_s_at	*PLAU*	0.203
203325_s_at	*COL5A1*	0.296	207173_x_at	*CDH11*	0.202
215076_s_at	*COL3A1*	0.295	213338_at	*TMEM158*	0.201
215446_s_at	*LOX*	0.293	209758_s_at	*MFAP5*	0.199
210495_x_at	*FN1*	0.291	202363_at	*SPOCK1*	0.195
201792_at	*AEBP1*	0.291	201148_s_at	*TIMP3*	0.195
216442_x_at	*FN1*	0.286	204051_s_at	*SFRP4*	0.193
212464_s_at	*FN1*	0.286	207172_s_at	*CDH11*	0.192
201852_x_at	*COL3A1*	0.286	202283_at	*SERPINF1*	0.191
212353_at	*SULF1*	0.285	209335_at	*DCN*	0.189
211719_x_at	*FN1*	0.285	204298_s_at	*LOX*	0.189
211161_s_at	*COL3A1*	0.283	219655_at	*C7orf10*	0.189
202403_s_at	*COL1A2*	0.278	219561_at	*COPZ2*	0.189
202766_s_at	*FBN1*	0.272	219773_at	*NOX4*	0.187
212354_at	*SULF1*	0.266	204464_s_at	*EDNRA*	0.186
219087_at	*ASPN*	0.260	200974_at	*ACTA2*	0.186
200665_s_at	*SPARC*	0.258	202273_at	*PDGFRB*	0.185
215646_s_at	*VCAN*	0.257	61734_at	*RCN3*	0.185
211571_s_at	*VCAN*	0.256	213139_at	*SNAI2*	0.183
202450_s_at	*CTSK*	0.254	220988_s_at	*AMACR*	0.182
206026_s_at	*TNFAIP6*	0.253	205713_s_at	*COMP*	0.181
202765_s_at	*FBN1*	0.247	201105_at	*LGALS1*	0.181
203876_s_at	*MMP11*	0.240	213869_x_at	*THY1*	0.180
212667_at	*SPARC*	0.239	202465_at	*PCOLCE*	0.174
222020_s_at	*HNT*	0.239	208851_s_at	*THY1*	0.174
206439_at	*EPYC*	0.235	209156_s_at	*COL6A2*	0.173
201069_at	*MMP2*	0.234	221447_s_at	*GLT8D2*	0.172
205479_s_at	*PLAU*	0.234	204114_at	*NID2*	0.171
206025_s_at	*TNFAIP6*	0.232	205991_s_at	*PRRX1*	0.171

^**a**^Affymetrix probe sets

**Figure 2:**
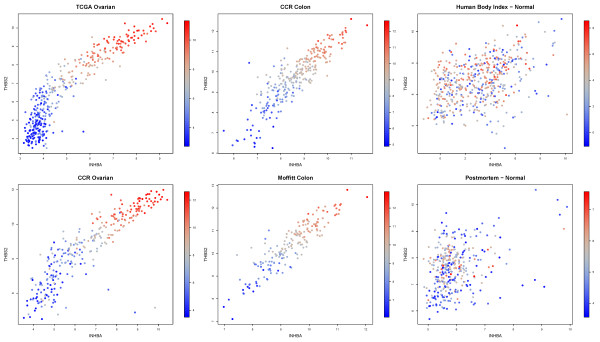
**Scatter plots confirming coexpression of INHBA, THBS2, COL11A1 in cancerous but not healthy data sets**. The expression of *COL11A1 *is color-coded). As shown, *COL11A1 *is consistently coexpressed with *INHBA *and *THBS2 *in cancerous, but not in noncancerous samples, two of which are shown on the right side.

Furthermore, contrary to all other genes, *COL11A1 *was uniquely *not *associated with any of these genes in noncancerous samples, further supporting the hypothesis that it can be used as a proxy for the MAF signature. Our results indicate that THBS2 and INHBA, top ranked in Table 4 except for collagens, are the most important players in the MAF mechanism. Figure 2 demonstrates this striking coexpression in data sets of cancer samples, but not in noncancerous samples, in the form of scatter plots. We have consistently validated this behavior in the cancerous and noncancerous data sets we tested.

**Figure 3:**
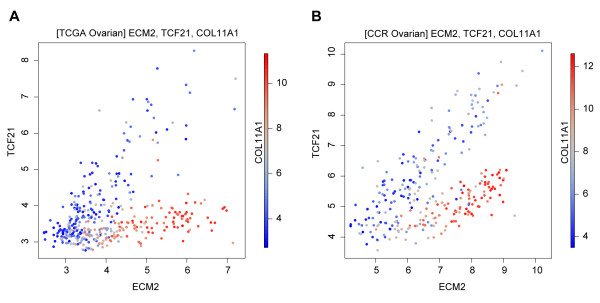
**Example of a synergistic pair of genes in two ovarian cancer data sets**.

As a second step, we identified gene pairs that are highest associated with *COL11A1 *jointly, but not individually, and therefore they would not appear in the previous list. For this task we ranked gene pairs according to their synergy [9] with *COL11A1*, using the computational method in [19], which could further facilitate biological discovery. For example, the scatter plots in Figure 3 show that genes *ECM2 *and *TCF21 *are jointly, but not individually, strongly associated with *COL11A1 *(p < 10^-6^, see Methods) in the two ovarian cancer data sets. Such findings are useful for developing biological hypotheses, e.g. in this particular case they suggest that in ovarian cancer the extracellular matrix protein 2 is associated with the MAF signature only when the *TCF21 *gene (a known mesenchymal-epithelial transition mediator) is downregulated.

The MAF signature exists even in non-epithelial cancers. Indeed, we confirmed that neuroblastoma also carries the MAF signature consistently associated with high stage: As shown in Additional file 5, none of 21 stage I samples have the signature (p = 4 × 10^-4^), based on the genes highest associated with *COL11A1*.

### MicroRNAs and Methylated sites

We only had miRNA and methylation data available for the TCGA ovarian data set. Using as measure the mutual information with *COL11A1*, we found many statistically significant miRNAs, among them hsa-miR-22 and hsa-miR-152, as well as differentially methylated genes, such as *SNAI1 *and *PRAME*, suggesting a particularly complex biological mechanism (correlation with the MAF phenotype led to essentially the same lists with lower significance). Table 5 contains a list of the miRNAs, while Table 6 contains a list of the methylated genes (multiple test corrected p < 10^-16 ^in both cases, see Methods). *SNAI1 *(*snail*) methylation is particularly important as the gene is known as one of the most important EMT-related transcription factors. Instead, the strongest MAF-associated transcription factor is AEBP1. Many of the other EMT-related transcription factors, such as SNAI2, TWIST1, and ZEB1 are often overexpressed in the MAF signature, but SNAI1 is not (and, at least in ovarian carcinoma in which we have methylation data, this is due to its differentially methylated status). We believe that the lack of SNAI1 expression is a key distinguishing feature of the MAF signature, in which we observed neither SNAI1 overexpression nor CDH1 (E-cadherin) downregulation, at least on the mRNA level.

**Table 5:** Top-ranked miRNAs in MAF signature in the TCGA ovarian cancer data set

miRNA	MI	Up/Down Regulated
hsa-miR-22	0.204	*Up*
hsa-miR-514-1|hsa-miR-514-2|hsa-miR-514-3	0.193	*Down*
hsa-miR-152	0.187	*Up*
hsa-miR-508	0.168	*Down*
hsa-miR-509-1|hsa-miR-509-2|hsa-miR-509-3	0.164	*Down*
hsa-miR-507	0.152	*Down*
hsa-miR-509-1|hsa-miR-509-2	0.147	*Down*
hsa-miR-506	0.146	*Down*
hsa-miR-509-3	0.144	*Down*
hsa-miR-214	0.128	*Up*
hsa-miR-510	0.116	*Down*
hsa-miR-199a-1|hsa-miR-199a-2	0.115	*Up*
hsa-miR-21	0.112	*Up*
hsa-miR-513c	0.108	*Down*
hsa-miR-199b	0.103	*Up*

All miRNAs have multiple test corrected p < 10^-16 ^in terms of their association with *COL11A1*.

**Table 6:** Top-ranked methylation sites in MAF signature in the TCGA ovarian cancer data set

Methylation site	MI	Hyper-/Hypomethylated
PRAME	0.223	*Hyper*
SNAI1	0.183	*Hyper*
KRT7	0.158	*Hyper*
RASSF5	0.157	*Hyper*
FLJ14816	0.155	*Hyper*
PPL	0.155	*Hyper*
CXCR6	0.153	*Hypo*
SLC12A8	0.148	*Hyper*
NFATC2	0.148	*Hyper*
HOM-TES-103	0.147	*Hypo*
ZNF556	0.147	*Hyper*
OCIAD2	0.146	*Hyper*
APS	0.142	*Hyper*
MGC9712	0.139	*Hyper*
SLC1A2	0.136	*Hyper*
HAK	0.131	*Hypo*
C3orf18	0.13	*Hyper*
GMPR	0.13	*Hyper*
CORO6	0.128	*Hyper*

All methylated genes have multiple-test corrected p < 10^-16 ^in terms of their association with *COL11A1*.

### Drug response

Significantly, we also found that, at least in ER negative breast cancer, the MAF signature is associated with resistance to neoadjuvant FEC. This was demonstrated in [20] where a stromal "metagene" signature of 50 genes was defined based on *DCN *(decorin). Although some of the MAF key genes (such as *COL11A1 *and *THBS2*) were not among these 50, the metagene signature used in that study has a significant intersection with the MAF signature and was found resistant to neoadjuvant chemotherapy. To compare the drug response performance of the *DCN *metagene set with that of the MAF signature, we used the top 50 genes of the MAF signature in terms of their association with *COL11A1 *from various cancers (the top genes shown in Table 4) in the same data set. Shown in Additional file 6 and Additional file 7 are two clustering heat maps of expression profiles, one with the MAF signature genes and one with the *DCN *metagene set, respectively. High expression of the MAF signature genes correlates with lack of response to therapy (identifying 14 samples out of which 12 have lack of response) more than high expression of the *DCN *metagene set (identifying 12 samples out of which 10 have lack of response), suggesting that the presence of the core genes of the MAF signature provide at least as good indicator of resistance to neoadjuvant chemotherapy. The reason for the drug resistance may simply be that the invasiveness sensed by the MAF signature does not allow the size or extent of the cancer to be reduced prior to surgery.

## Discussion

A direct clinical application of these findings is the development of a high-specificity invasion-sensing biomarker product detecting coordinated overexpression of a few top-ranked genes, such as *COL11A1*, *INHBA, and THBS2*, as shown in the scatter plots of Figure 2. A positive result in seemingly low-stage primary tumors will indicate that the disease has obtained the stromal signature and thus has already reached an invasive stage. As described above, the same product can also be used to predict resistance to neoadjuvant chemotherapy.

Of course, the most significant clinical application would be to develop metastasis-inhibiting therapeutics using targets deduced from the biological knowledge provided by the MAF signature. Our top ranked genes strongly suggest that they are produced by myofibroblasts or myofibroblast-like cells activated by activin A-induced TGF-β signaling and leading to some form of altered proteolysis [21], which results in extracellular matrix remodeling. Supporting this hypothesis are the facts that both INHBA and THBS2 are involved in TGF-β signaling: Activin A (INHBA homodimer) is a TGF-β superfamily member (ligand) and THBS2 inhibits activation of TGF-β by THBS1, which is also present in the MAF signature. Remarkably, activin A is already known to facilitate fibroblast-mediated collagen gel contraction [22]. The role of gene *LRRC15 *(aka *LIB*) appears important but unclear, though it has already been recognized as promoting migration through the extracellular matrix [23]. Versican (VCAN) is an extracellular matrix proteoglycan already known to play a role in metastasis, while MMP11 is one of several matrix metalloproteinases involved in the breakdown of extracellular matrix.

Although each of the MAF signature molecules could serve as a potential therapeutic target, the hypothesis that activin A signaling is at the heart of the MAF mechanism immediately suggests that follistatin (activin-binding protein) could serve as a metastasis inhibitor, which is exactly what recent research [24] indicates. Specifically, lung cancer cell lines transfected with follistatin and injected into immunodeficient mice markedly inhibited metastasis compared with non-transfected cell lines, but the authors of the study recognize that the role of follistatin in cancer metastasis is totally unknown [25]. Our work provides an explanation and suggests that the same could be true for other cancers as well. Further support is provided by the fact that follistatin virtually abolished the fibroblast-mediated collagen gel contraction mentioned earlier [22].

There are several reasons that the core MAF signature has not yet been discovered as a multi-cancer metastasis-associated signature. First, it is essential to identify the correct phenotypic staging threshold recognizing that the signature only exists in a subset of tumors that exceed that particular stage. Indeed, if the threshold in breast cancer was put between stage I and stage II, or between stage II and stage III, rather than between *in situ *and stage I, the signature would not be apparent. Second, each cancer type may have its own additional features accompanying the MAF signature. For example, in ovarian cancer it is accompanied by sharp downregulation of genes *COLEC11*, *PEG3 *and *TSPAN8*, which is not the case in other cancers. Indeed, the main contribution of our work is the identification of the common multi-cancer "core" signature, from which a universal metastasis-associated biological mechanism can be identified. Third, the MAF signature may be reversible, perhaps as a result of the disappearance of many of the stromal cells in the mature desmoplastic stroma when it is replaced by "acellular" matrix [6]. The presence of the signature in high-stage samples may even paradoxically be associated with longer survival if its reversal is required for further distant metastases (see below).

An important topic of further research is the determination of the precise biological event of interaction of cancer cells with the microenvironment that gives rise to the stromal MAF signature and associated invasiveness. Because of the recognized similarities with the mechanisms of wound healing [26], it is likely that this event uses existing wound healing response pathways. For example, it appears to occur very early in breast cancer, late in ovarian cancer, and never in glioblastoma (which is reasonable, because glioblastoma metastasizes extremely rarely). The late appearance of the MAF signature in ovarian cancer and its presence in omental metastases can be explained by the fact that ovarian cancer initially progresses by disseminating locally across mesothelial surfaces and that, contrary to hematogenously metastasizing tumors, initial metastasis is probably carried by the physiological movement of peritoneal fluid to the peritoneum and omentum [27].

Several of the top-ranked genes in the MAF signature (such as thrombospondins, decorin, INHBA itself) are known to be potent anti-angiogenesis mediators. The reversal of the MAF signature would thus facilitate further metastatic dissemination to distant sites. In other words, (a) the desmoplastic MAF signature and (b) angiogenesis, are two independent biological events. The former appears to be based on activin A signaling, as several of the MAF proteins in addition to INHBA are also known inhibitors of the standard TGF-β ligand. The reversal of the MAF signature would allow the standard ligand to take over in TGF-β signaling, and may thus facilitate further metastasis. These observations provide explanations for the seemingly contradictory observed roles of TGF-β signaling inhibiting early cancer but facilitating metastasis.

The possible reversibility of the MAF signature leads to the intriguing hypothesis that perhaps all metastases have, at some point temporarily been there, which explains why we only observe it in a subset of them. This would be particularly exciting, because in that case any metastasis-inhibiting therapeutic intervention targeting the MAF mechanism would be widely applicable to low-stage tumors.

## Conclusions

In conclusion, we have shown that, using purely computational analysis of publicly available biological information, systems biology has revealed the core of a multi-cancer metastasis-associated gene expression signature. In the near future, a vast amount of additional information will become available, including next generation sequencing, miRNA and methylation information for many cancers, which will allow additional computational research building on this work and clarifying the details of the underlying invasion-associated complex biological process.

## Competing interests

The authors declare that they have no competing interests.

## Authors' contributions

DA and VV conceived of and supervised the study. HK and JW carried out the experiments. DA drafted the manuscript. All authors participated in the study, read and approved the final manuscript.

## Pre-publication history

The pre-publication history for this paper can be accessed here:

http://www.biomedcentral.com/1755-8794/3/51/prepub

## Supplementary Material

Additional file 1 **Heat map of TCGA ovarian cancer data set**. This file contains the result of hierarchical clustering for the TCGA ovarian cancer data set using a particular gene set (see text). Click here for file

Additional file 2 **Implementation of the EVA algorithm**. This file contains the description of a low-complexity implementation of the EVA algorithm. Click here for file

Additional file 3 **List of genes resulting from EVA algorithm**. This file contains the commonly significant probe sets in four ovarian and colorectal cancers data sets. Click here for file

Additional file 4 **Listing of genes in 9 cancer data sets**. This file contains a color-coded list of genes in various cancers ranked in terms of the mutual information with *COL11A1*. Click here for file

Additional file 5 **Heat map of neuroblastoma data set**. This file contains the result of hierarchical clustering for the neuroblastoma data set (GSE3960) using the MAF signature genes. Click here for file

Additional file 6 **Heat map of breast cancer data set using MAF signature genes**. This file contains the result of hierarchical clustering for the breast cancer data set (GSE4779) using the MAF signature genes. Click here for file

Additional file 7 **Heat map of breast cancer data set using *DCN *metagene set**. This file contains the result of hierarchical clustering for the breast cancer data set (GSE4779) using the *DCN *metagene set. Click here for file
