# Supplementary figures and images for: Multi-cancer computational analysis reveals invasion-associated variant of desmoplastic reaction involving INHBA, THBS2 and COL11A1
Source: BMC Med Genomics. 2010 Nov 3;3:51. doi: 10.1186/1755-8794-3-51 (PMC2988703; doi:10.1186/1755-8794-3-51)

Tumor Stage

|      |
|------|
| null |
| IA   |
| IIA  |
| IC   |
| IIA  |
| IIB  |
| IIC  |
| IIIA |
| IIIB |
| IIIC |
| IV   |

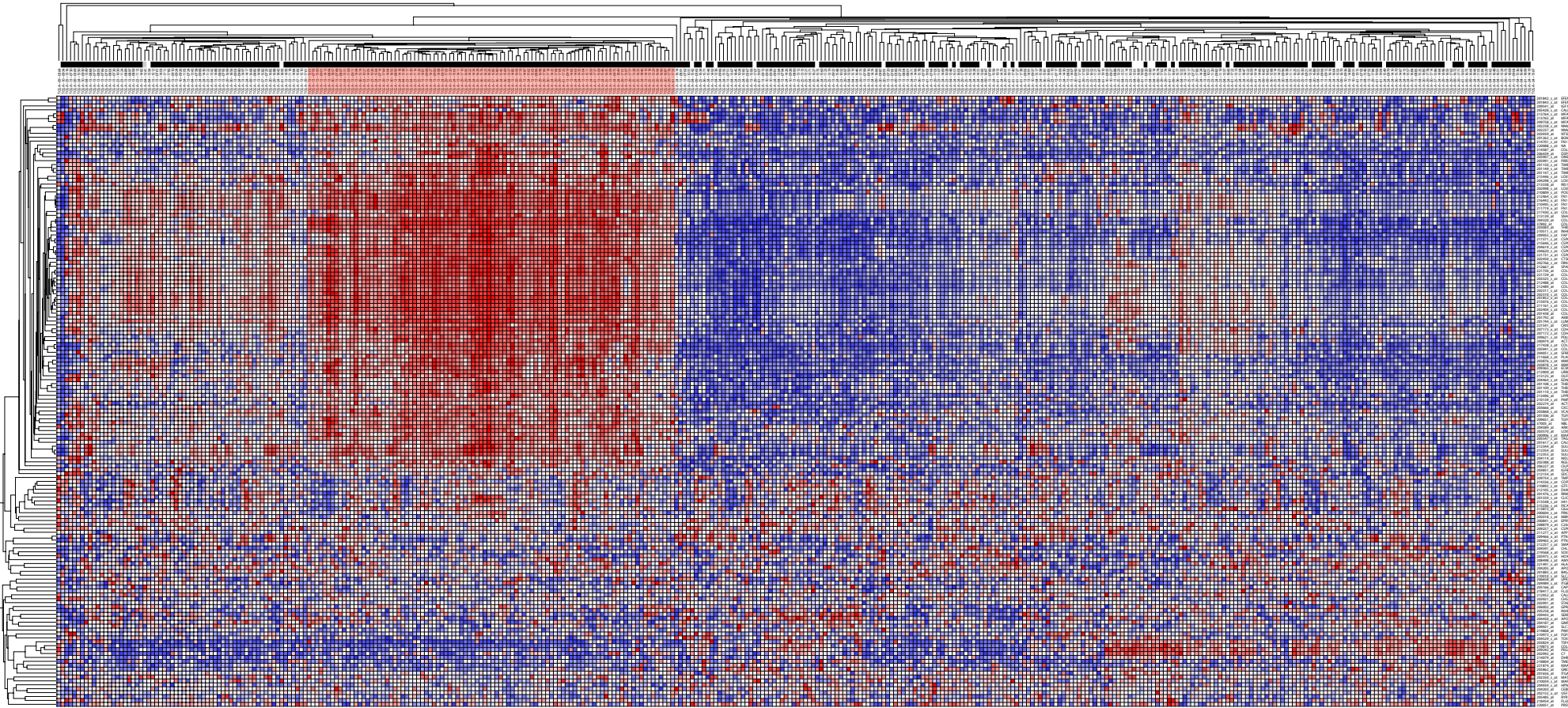

Supplement: Additional file 1 — Heat map of TCGA ovarian cancer data set. This file contains the result of hierarchical clustering for the TCGA ovarian cancer data set using a particular gene set (see text). [file 1755-8794-3-51-S1.PDF]

INSS Stage

- NA  
1  
1\*  
3  
4

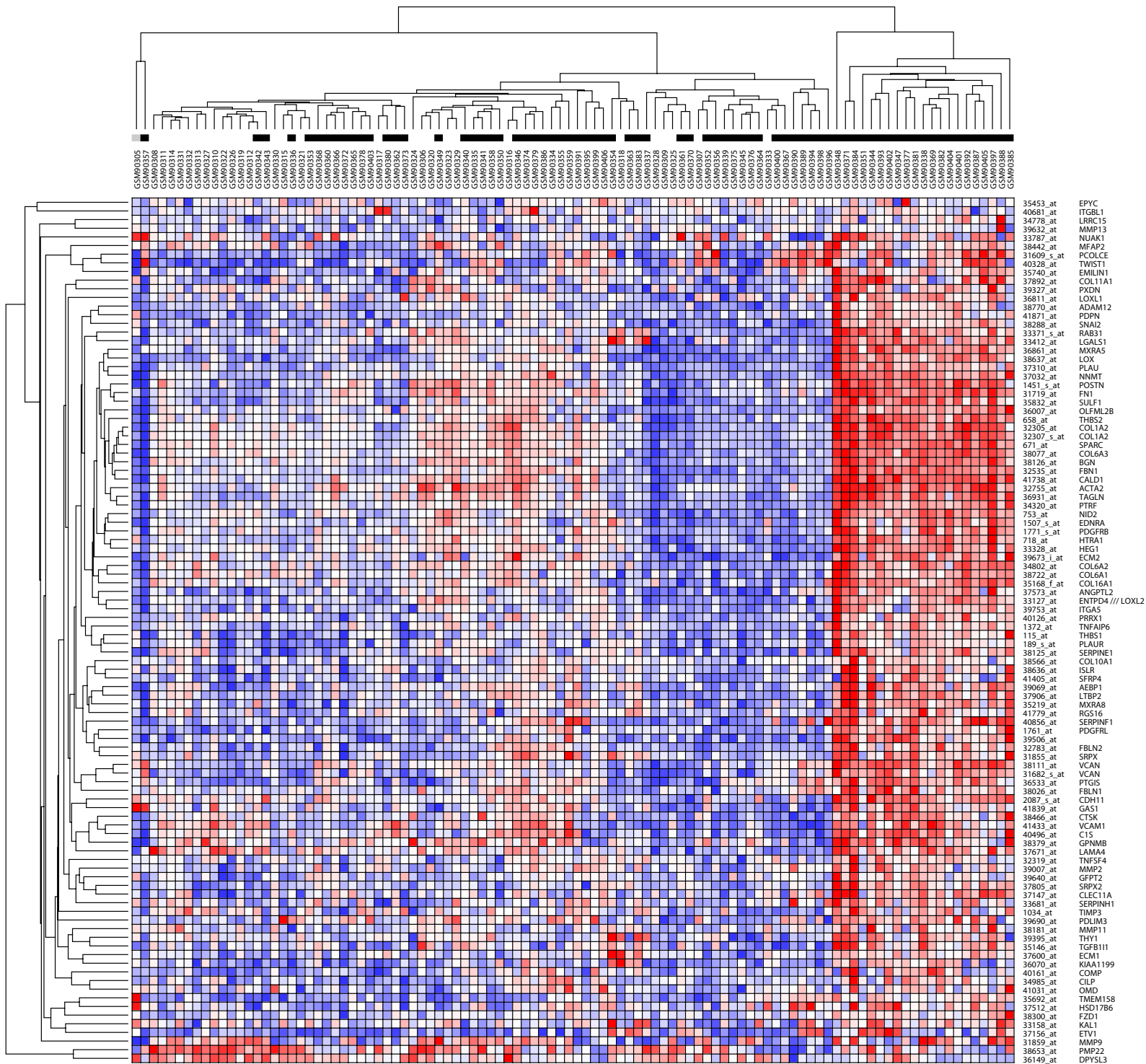

Supplement: Additional file 5 — Heat map of neuroblastoma data set. This file contains the result of hierarchical clustering for the neuroblastoma data set (GSE3960) using the MAF signature genes. [file 1755-8794-3-51-S5.PDF]

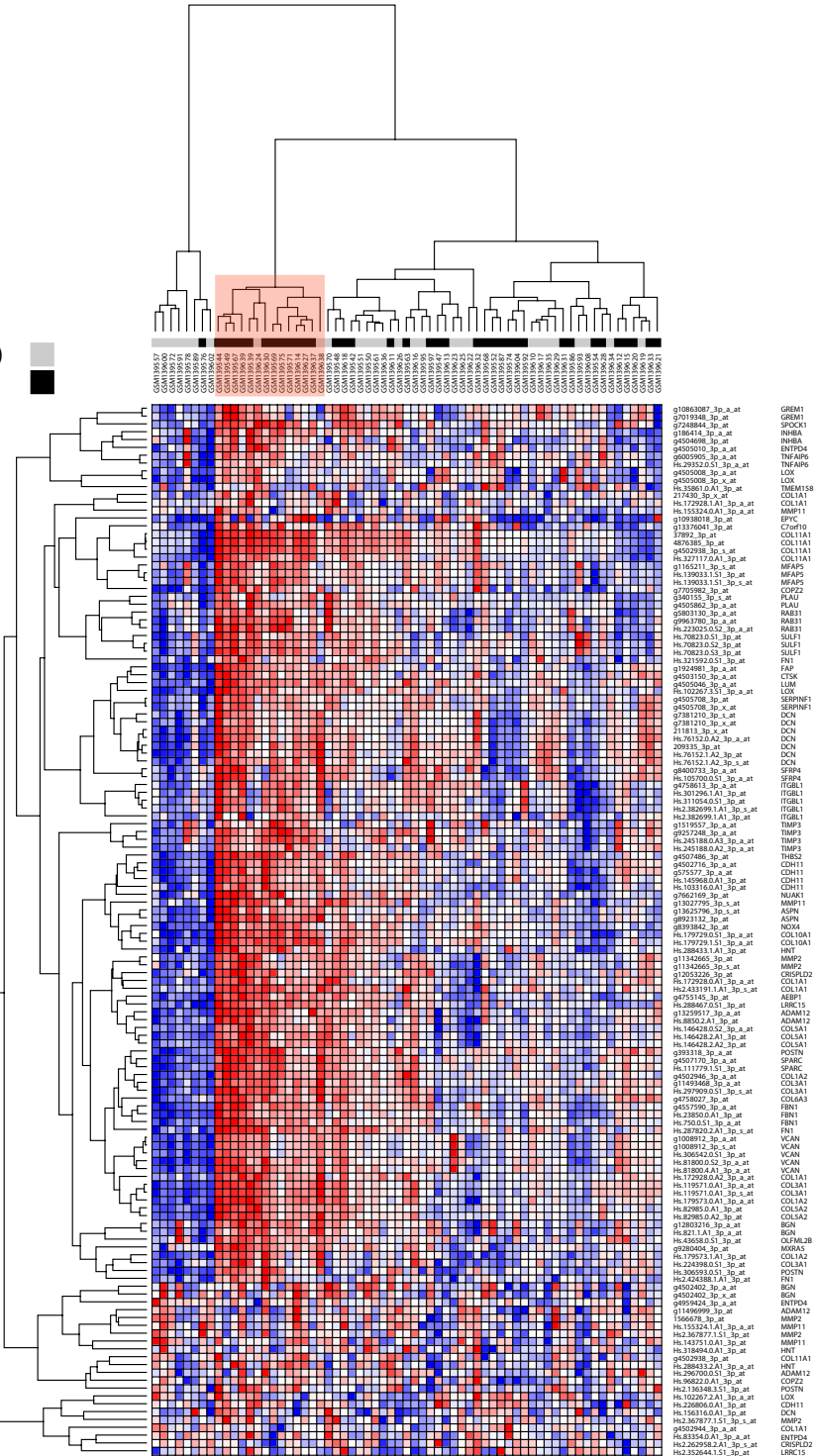

Supplement: Additional file 6 — Heat map of breast cancer data set using MAF signature genes. This file contains the result of hierarchical clustering for the breast cancer data set (GSE4779) using the MAF signature genes. [file 1755-8794-3-51-S6.PDF]

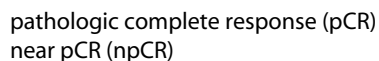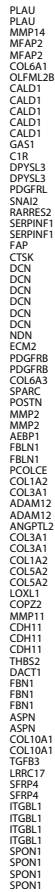

Supplement: Additional file 7 — Heat map of breast cancer data set using DCN metagene set. This file contains the result of hierarchical clustering for the breast cancer data set (GSE4779) using the DCN metagene set. [file 1755-8794-3-51-S7.PDF]
