# Supplementary material for: Multi-cancer computational analysis reveals invasion-associated variant of desmoplastic reaction involving INHBA, THBS2 and COL11A1
Source: BMC Med Genomics. 2010 Nov 3;3:51. doi: 10.1186/1755-8794-3-51 (PMC2988703; doi:10.1186/1755-8794-3-51)
Supplement: Additional file 2 — Implementation of the EVA algorithm. This file contains the description of a low-complexity implementation of the EVA algorithm. [file 1755-8794-3-51-S2.PDF]

# Additional file 2

# Implementation of the EVA algorithm

The algorithm dynamically "builds" the cumulative hypergeometric distribution for each probe set, as follows: Given a data set with a high-stage samples and b-low stage samples, the idea is to construct an $(a+1)\times(b+1)$ table of the hypergeometric probabilities corresponding to all possible subsets of the samples. Then, for each probe set, the samples are sorted according to the expression value of the probe set. This ordering results in a path through the table from the bottom left corner to the top right corner, moving either up or to the right for each sample. At each step in the path, the cumulative probability of encountering the observed number of high stage samples or more is computed by summing the entries diagonally down and to the right of the current cell, including the current cell itself. The low-complexity algorithm is best demonstrated with a visual example:

| Low Stage Count | | | | | | |
|---|---|---|---|---|---|---|
| **3** | 0.018 | 0.071 | 0.179 | 0.357 | 0.625 | 1.000 |
| **2** | 0.107 | 0.268 | 0.429 | 0.536 | 0.536 | 0.375 |
| **1** | 0.375 | 0.536 | 0.536 | 0.429 | 0.268 | 0.107 |
| **0** | 1.000 | 0.625 | 0.357 | 0.179 | 0.071 | 0.018 |
| | **0** | **1** | **2** | **3** | **4** | **5** |

*High Stage Count*

in which the data set has three low stage samples and five high stage samples in total. Each probe set results in a path through this table, and an example path is displayed here in gray. Letting *1* correspond to a high stage sample and *0* correspond to a low

stage sample, this example probe set results in the path *111001011*. For the cell in blue, corresponding to the sub-path *111001*, the probability of encountering this many high stage samples or more is computed by summing the three probabilities diagonally down and to the right of the blue cell (including itself). In this case, the probability is quite high (82.2%). This cumulative probability is computed for every step along the path, and the minimum of these is the output of the EVA algorithm. The pseudo-code for the EVA algorithm is as follows:

```
Input:
Let n be the number of samples.
Let a be the number of high stage samples.
Let b be the number of low stage samples.
Let s be the array of phenotype labels sorted by this probe set's
expression level
(Note: a + b = n)

Algorithm:
// Step 1 – Build the table of hypergeometric probabilities.
// This step need only be run once for the entire data set.
Define c as an array with (a+1) rows and (b+1) columns.
For x from 0 to a
  For y from 0 to b
    If x = 0 and y = 0
       c[x][y] = 0
    Else
       If (y > 0)
         c[x][y] = c[x][y] + c[x][y - 1] * (b - y + 1) / ((b - y + 1) +
(a - x))
       End if
       If (x > 0)
         c[x][y] = c[x][y] + c[x - 1][y] * (a - x + 1) / ((a - x + 1) +
(b - y))
       End If
    End If
  End For
End For
// Step 2 – Compute the cumulative hypergeometric probability for the
given sequence.
Define x = 0
Define y = 0
Define bestP = 1
For i from 1 to n
  If (s[i] = 1)
    x = x + 1
  Else
    y = y + 1
  End If
  Define p = 0
  For j from 0 to y
    If (x + y - j <= a)
      p = p + c[x + y - j][j]
    End If
```

```
  End For
  If (p < bestP)
    bestP = p
  End If
End For
```

**Output:**

```
  -log10(bestP)
```
